# Monitoring aerobic capacity in cancer survivors using self-reported questionnaires: criterion validity and responsiveness

**DOI:** 10.1186/s41687-023-00613-8

**Published:** 2023-07-19

**Authors:** Anouk T.R. Weemaes, Renske Meijer, Milou Beelen, Martijn van Hooff, Matty P. Weijenberg, Antoine F. Lenssen, Lonneke V. van de Poll-Franse, Hans H.C.M. Savelberg, Goof Schep

**Affiliations:** 1 Department of Physical Therapy, Maastricht University Medical Center+, P.O. Box 5800, Maastricht, AZ 6202 The Netherlands; 2 Department of Epidemiology, Care and Public Health Research Institute (CAPHRI), Faculty of Health Medicine and Life Sciences, Maastricht University, Maastricht, The Netherlands; 3 Department of Sports and Exercise, Máxima Medical Center, Veldhoven, The Netherlands; 4 Department of Nutrition and Movement Sciences, NUTRIM School of Nutrition and Translational Research in Metabolism Faculty of Health Medicine and Life Sciences, Maastricht University, Maastricht, The Netherlands; 5 Department of Human Biology, School of Nutrition and Translational Research in Metabolism (NUTRIM), Faculty of Health Medicine and Life Sciences, Maastricht University, Maastricht, The Netherlands; 6 Department of Epidemiology, GROW School for Oncology and Reproduction, Faculty of Health Medicine and Life Sciences, Maastricht University, Maastricht, the Netherlands; 7 Department of Research and Development, Netherlands Comprehensive Cancer Organization, Utrecht, The Netherlands; 8 Division of Psychosocial Research & Epidemiology, The Netherlands Cancer Institute, Amsterdam, The Netherlands; 9 Department of Medical and Clinical Psychology, Tilburg University, Tilburg, The Netherlands

**Keywords:** Psychometric properties, Cardiopulmonary exercise test, Exercise rehabilitation, Cardiorespiratory fitness, Patient-reported outcome measures

## Abstract

**Background:**

Evaluating the criterion validity and responsiveness of the self-reported FitMáx©-questionnaire, Duke Activity Status Index (DASI) and Veterans Specific Activity Questionnaire (VSAQ) to monitor aerobic capacity in cancer survivors.

**Methods:**

Cancer survivors participating in a 10-week supervised exercise program were included. The FitMáx©-questionnaire, DASI, VSAQ and a cardiopulmonary exercise test (CPET) were completed before (T_0_) and after (T_1_) the program. Intraclass correlation coefficients (ICC) were calculated between VO_2peak_ estimated by the questionnaires (questionnaire-VO_2peak_) and VO_2peak_ measured during CPET (CPET-VO_2peak_), at T_0_ to examine criterion validity, and between changes in questionnaire-VO_2peak_ and CPET-VO_2peak_ (ΔT_0_-T_1_) to determine responsiveness. Receiver operating characteristic (ROC) analyses were performed to examine the ability of the questionnaires to detect true improvements (≥ 6%) in CPET-VO_2peak_.

**Results:**

Seventy participants were included. Outcomes at T_1_ were available for 58 participants (83%). Mean CPET-VO_2peak_ significantly improved at T_1_ (Δ1.6 mL·kg^− 1^·min^− 1^ or 8%). Agreement between questionnaire-VO_2peak_ and CPET-VO_2peak_ at T_0_ was moderate for the FitMáx©-questionnaire (ICC = 0.69) and VSAQ (ICC = 0.53), and poor for DASI (ICC = 0.36). Poor agreement was found between ΔCPET-VO_2peak_ and Δquestionnaire-VO_2peak_ for all questionnaires (ICC 0.43, 0.19 and 0.18 for the FitMáx©-questionnaire, VSAQ and DASI, respectively). ROC analysis showed that the FitMáx©-questionnaire was able to detect improvements in CPET-VO_2peak_ (area under the curve, AUC = 0.77), when using a cut-off value of 1.0 mL·kg^− 1^·min^− 1^, while VSAQ (AUC = 0.66) and DASI (AUC = 0.64) could not.

**Conclusion:**

The self-reported FitMáx©-questionnaire has sufficient validity to estimate aerobic capacity in cancer survivors at group level. The responsiveness of the FitMáx©-questionnaire for absolute change is limited, but the questionnaire is able to detect whether aerobic capacity improved. The FitMáx©-questionnaire showed substantial better values of validity and responsiveness compared to DASI and VSAQ.

## Background

Cancer and its medical treatment often lead to impairments in aerobic capacity and consequently decreased physical functioning and health-related quality of life. Literature suggests that low aerobic capacity is associated with increased risks for cancer-recurrence and all-cause and cancer-related mortality [1, 2]. Therefore, it is worrying that cancer survivors experience a longstanding decline in aerobic capacity of 5–22% during the course of their treatment [3, 4]. This decline in aerobic capacity can be countered or prevented, and it is well-known that physical exercise is an effective way to do so [5, 6].

The criterion standard to evaluate aerobic capacity is measuring peak oxygen uptake (VO_2peak_) during an incremental maximal exercise test with respiratory gas analysis, also referred to as a cardiopulmonary exercise test (CPET) [7]. Measuring VO_2peak_ is of great additional value for pre-operative risk-screening, personalized exercise prescription and monitoring aerobic capacity in patients with cancer [8, 9]. Moreover, CPET is used for exercise pre-participation health screening and to determine the underlying cause of exercise limitation [9, 10]. However, performing CPET is costly, time-consuming, a burden to the patient and requires costly advanced equipment and medical supervision [9]. In many clinical circumstances the main aim is to assess aerobic capacity, without underlying diagnostic question on exercise limitation. Patient-reported outcome measures (PROMs), such as self-reported questionnaires, could be a useful alternative to estimate and monitor aerobic capacity in these settings where a CPET is not feasible or necessary.

The Duke Activity Status Index (DASI) and Veterans Specific Activity Questionnaire (VSAQ) are self-reported questionnaires which are often used in clinical healthcare for the assessment of aerobic capacity in patients [11, 12]. The DASI was developed to assess physical functioning in cardiovascular patients and shows good validity compared to VO_2peak_ measured during CPET (CPET-VO_2peak_) when administered by an interviewer, and moderate validity when self-reported [11]. In a recent study with patients scheduled for major cancer surgery, VO_2peak_ estimated using the DASI (DASI-VO_2peak_) showed substantial bias with wide 95% limits of agreement (95%-LoA) when compared to CPET-VO_2peak_ [13]. The VSAQ was developed to estimate aerobic capacity in American veterans describing activities of increasing Metabolic Equivalent of a Task (MET) and showed a moderate correlation with METs derived from CPET [12]. One MET is considered equal to 3.5 mL·kg^− 1^·min^− 1^ and can be used interchangeable with VO_2peak_ [14]. In a more recent study with healthy adults, VO_2peak_ estimated using the VSAQ (VSAQ-VO_2peak_) also showed considerable bias with wide 95%-LoA [15]. Although VSAQ and DASI showed a significant correlation with measured VO_2peak_, agreement was suboptimal. Besides, both questionnaires were developed and validated in an American population. A major drawback of the VSAQ is the use of activities, such as basketball and cross-country skiing, which are not practiced globally [16].

More recently, the FitMáx©-questionnaire, hereafter called FitMáx, was developed as a self-reported questionnaire to estimate VO_2peak_ (FitMáx-VO_2peak_) in the general Dutch population. FitMáx-VO_2peak_ is based on the self-reported maximum capacity of walking, stair climbing, and cycling combined with age, sex, and body mass index (BMI). In a recent study, the FitMáx showed a strong intraclass correlation (ICC = 0.93) with CPET-VO_2peak_, and acceptable bias (-0.24 with 95%-LoA − 9.23–8.75), in a heterogeneous group of 228 patients (with lung, cardiac and oncologic diseases) and athletes. The results for FitMáx were compared with DASI (ICC = 0.62, bias of 3.32 with 95%-LoA − 14.81–21.44) and VSAQ (ICC = 0.87, bias of 3.44 with 95%-LoA − 10.11–16.98) in the same population and showed better agreement with CPET-VO_2peak_ [17].

The clinical usefulness and applicability of PROMs depend on several measurement properties including validity, responsiveness and reliability. Assessing the responsiveness of an instrument is important to determine whether it is able to detect changes over time. However, no studies regarding the responsiveness of these self-reported questionnaires were performed before. Therefore, the aim of this study was to assess and compare the (1) population specific criterion validity and (2) responsiveness of VO_2peak_ predicted by FitMáx, DASI and VSAQ as self-reported questionnaires, to evaluate aerobic capacity in cancer survivors who participated in a 10-week supervised exercise program.

We hypothesized the population specific agreement between CPET-VO_2peak_ and FitMáx-VO_2peak_ at T_0_ to be moderate-to good, with an ICC of > 0.70 [17–19]; and the ICC between change over time in CPET-VO_2peak_ and FitMáx-VO_2peak_ to be between 0.40 and 0.60 [20, 21]. Furthermore, the ability of the FitMáx to discriminate between participants who did or did not improve in aerobic capacity was expected to be moderate. As such, the area under the curve (AUC) of the receiver operating characteristic curve (ROC-curve) was expected to be in the range of 0.60–0.80 [18]. Lastly, looking at the results of previous studies, the validity and responsiveness of FitMáx-VO_2peak_ in this population are expected to be better compared to the validity and responsiveness of the DASI-VO_2peak_ and VSAQ-VO_2peak_, which are expected to show poor-to moderate agreement with CPET-VO_2peak_ (ICC < 0.70) [11, 17, 22].

## Methods

### Setting

Patients who were scheduled to participate in a supervised exercise program as part of usual-care multidisciplinary oncology rehabilitation, were prospectively recruited at the Department of Physical Therapy of the Maastricht University Medical Center (MUMC+) between January 2021 and December 2021.The multidisciplinary rehabilitation program consisted of a 10-week supervised physical exercise program, supplemented with psychological and/or occupational therapy, when indicated. The exercise program consisted of combined endurance and resistance training as described elsewhere [23]. Data collection procedures were in compliance with the Declaration of Helsinki [24] and were approved by the medical ethics committee of the MUMC+ (registration number METC 2020–2300). This study was reported according to the Consensus-Based Standards for the Selection of Health Measurement Instruments (COSMIN) guidelines [25]. The study was registered as NL8568 in the Netherlands Trial Register (https://trialsearch.who.int).

### Participants

Patients were eligible to participate in the rehabilitation program when they were suffering from physical and psychosocial complaints and/or fatigue due to cancer (treatments). Patients were excluded from participation when they were unable to perform basic activities of daily living (e.g. walking) and suffered from disabling comorbidities that seriously hamper physical exercise [23]. Within two weeks before the start (T_0_) and after the 10-week exercise program (T_1_) a CPET was conducted as part of usual care. Patients were included in this study when they were willing to complete three self-reported questionnaires during both CPET consultations and gave written informed consent for the use of their questionnaire and CPET data. Patients who were unable to read and understand the questionnaires, or did not show signs of voluntary exhaustion during the CPET at T_0_ (e.g. due to injuries or joint complaints) were excluded from the study.

### Test procedures

Anthropometric measurements were conducted before the CPET. After pre-test instructions, baseline cardiopulmonary values were collected during a 2-minute rest period while seated at the cycle ergometer (Lode Corival, Lode BV, Groningen, The Netherlands). After the rest period, the participant completed a 3-minute warm-up phase of unloaded cycling. Subsequently, the work rate started to increase by an incremental maximal ramp protocol adjusted to the patients’ self-reported physical activity level (assessed by the sports physician independently from the questionnaire results), aimed to reach a maximal effort within 8‒12 min [26, 27]. At T_1_, the same ramp protocol was applied for CPET as at T_0_. Participants were instructed to keep cycling until exhaustion, with a pedaling frequency of at least 60 rotations per minute. The protocol continued until the patient stopped cycling or pedaling frequency fell below 60 rotations per minute, despite verbal encouragement. Continuous breath-by-breath analysis was obtained during the test using a ergospirometry system (Vyntus CPX, Vyaire Medical, Mettawa, United States) calibrated for respiratory gas analysis and volume measurements. Peak exercise was defined as the point where the pedaling frequency dropped below 60 rotations per minute. Voluntary exhaustion was considered to be achieved when participants showed clinical signs of intense effort (e.g., unsteady biking, sweating or clear unwillingness to continue exercising). True maximal effort was considered to be reached if one of the two following criteria was met: (i) percentage of age related predicted maximal heart rate and (ii) age related peak respiratory exchange rate (RER_peak_) [28, 29]. Participants were blinded for test outcomes during both test moments and for questionnaire answers at T_0_, during T_1_ measurements. Moreover, researchers were blinded for questionnaire data during the CPET and for test outcomes at T_0_ during the CPET at T_1_. CPET outcomes were analyzed by a trained researcher. Oxygen uptake (VO_2_) and RER values were averaged over 30 s at peak exercise. The VO_2_ at the anaerobic threshold (VO_2AT_) was determined as described elsewhere [30].

### Questionnaires

On the same day, shortly before the CPET subjects were asked to complete the DASI, VSAQ and FitMáx as self-reported questionnaires. The DASI consists of twelve dichotomous questions, of which weighted scores are used in an algorithm to estimate the VO_2peak_ [11]. The VSAQ is a single-answer 13-point scale describing activities of increasing intensity. The VSAQ score and age were used to estimate VO_2peak_, according to guidelines of the questionnaire [12]. The FitMáx consists of three single-answer, multiple-choice questions assessing the maximum capacity of walking, stair climbing, and cycling on a 14-, 11- and 12-point scale, respectively. Based on the weighted score of the FitMáx combined with sex, age (in whole years) and BMI, VO_2peak_ was estimated [17]. The ability of the current study population to complete the FitMáx was assessed using three additional questions on a scale 1‒10 for the questions about walking, stair climbing and cycling capacity separately, in which 1 indicates “I cannot estimate properly” and 10 indicates “I can estimate properly”.

### Statistical analysis

A sample size estimation was performed using PASS 2008 [31], in which a sample size of n = 55 was determined to achieve a two-way 95% confidence interval with an expected correlation of r = 0.60 (0.40–0.75). This in in line with the minimum of 50 participants as recommended in the COSMIN guidelines [25]. Statistical analyses were performed using SPSS version 23.0 [32]. Continuous variables were checked for normality using histograms and Q-Q plots. Continuous variables are presented as mean ± standard deviation (SD) in case of normal distribution or as median and interquartile range otherwise. Categorical variables are expressed as frequencies with percentages. Mean changes in outcomes between T_0_ and T_1_ were reported with 95%-CI. When the 95%-CI did not include zero, the mean change was considered statistically significant. Criterion validity and responsiveness were determined using ICC (two-way random, absolute agreement), with corresponding 95%-CI and standard error of the estimate (SEE). Criterion validity of the FitMáx, DASI and VSAQ was evaluated for all participants at T_0_, by quantifying the agreement between CPET-VO_2peak_ and VO_2peak_ estimated using the questionnaires (questionnaire-VO_2peak_). Furthermore, Bland-Altman analysis was conducted with calculation of bias and 95%-LoA to assess the agreement between CPET-VO_2peak_ and questionnaire-VO_2peak_ and to determine whether mean differences between both values, are dependent on the size of the CPET-VO_2peak_. Proportional bias was assessed using linear regression between the means and the differences of CPET-VO_2peak_ and questionnaire-VO_2peak_. P-values of < 0.05 were considered statistically significant. In case of proportional bias, the ratio of questionnaire-VO_2peak_ to CPET-VO_2peak_ was calculated for each subject and plotted to the average of the two values with corresponding 95%-LoA, as suggested by Bland and Altman [33]. To evaluate the responsiveness of the FitMáx, DASI and VSAQ, the ICC and SEE were calculated between the absolute change in CPET-VO_2peak_ (ΔCPET-VO_2peak_) and questionnaire-VO_2peak_ (Δquestionnaire-VO_2peak_) between T_0_ and T_1_, for participants who completed both exercise tests. As a secondary analysis, the FitMáx-VO_2peak_ without cycling was included for analysis as well, since it was expected that not all participants cycle regularly (on a regular bicycle without electronic support).

If the responsiveness to estimate ΔCPET-VO_2peak_ was insufficient (ICC < 0.50), ROC-curves were plotted between the dichotomized ΔCPET-VO_2peak_ (improvement vs. no improvement) and the Δquestionnaire-VO_2peak_ to assess whether the questionnaires at least were able to detect improvement in CPET-VO_2peak_ [19–21] The minimal detectable change for improvement in CPET-VO_2peak_ was defined as a relative increase of ≥ 6% [34]. The AUC of the ROC-curve with corresponding 95%-CI was calculated to evaluate the ability of the questionnaires to detect a true improvement in CPET-VO_2peak_ of ≥ 6% over time. Since both sensitivity and specificity were considered equally important, the value at which the product of both is maximized was chosen as the optimal cut-off value to indicate an improvement in CPET-VO_2peak_ [35]. Sensitivity, specificity, and predictive values (%) were calculated for the cut-off values of the questionnaires.

## Results

### Participants

**Fig. 1:**
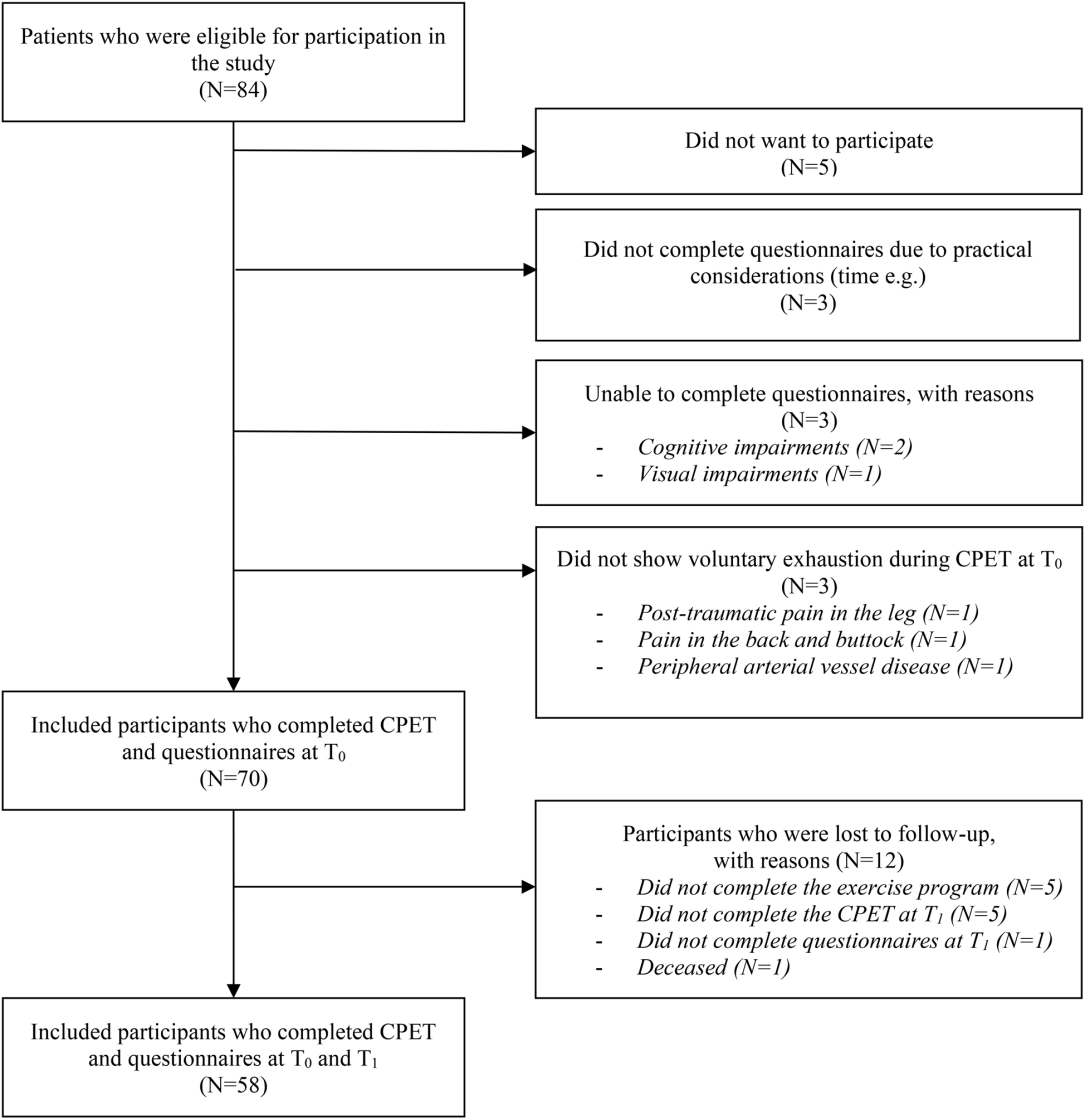
Participant inclusion flowchart Abbreviations: CPET, cardiopulmonary exercise test; n, number of subjects

Of the 84 patients who were eligible to participate in the study, 70 participants (83%) were included for analysis (15 men and 55 women). Twelve participants (17%) were lost to follow-up, because they did not complete any of the questionnaires and/or the CPET at T_1_, for several reasons. Outcome measures at T_1_ were available for 58 participants (83%) (see Fig. 1). Mean age at T_0_ was 53.2 ± 12.8 years and breast cancer was the most common diagnosis (39%). Surgery, chemotherapy and radiotherapy were the most commonly received treatments and approximately half of the participants were still receiving medical treatment during the study. Three of them (4%) were still receiving chemotherapy (Table 1).

**Table 1:** Patient characteristics at baseline (T_0_)

Characteristic	Participants who completed tests and questionnaires at T_0_ (N = 70)	Participants who completed tests and questionnaires at T_0_ and T_1_ (N = 58)
*Anthropometric data*		
Sex		
Male	15 (21%)	9 (16%)
Female	55 (79%)	49 (85%)
Age (years)	53.2 (± 12.8)	54.1 (± 11.6)
Body Mass Index (kg·m^− 2^)	27.6 (± 5.6)	27.5 (± 5.5)
*Cancer type*		
Breast cancer	27 (39%)	25 (43%)
Hematologic cancer	12 (17%)	7 (12%)
Cervix carcinoma	6 (9%)	4 (7%)
Lung cancer	5 (7%)	4 (7%)
Melanoma	4 (6%)	3 (5%)
Other	16 (23%)	15 (26%)
*Metastasis*		
No metastasis	37 (53%)	31 (53%)
Lymphatic metastasis	23 (33%)	19 (33%)
Bone metastasis	4 (6%)	3 (5%)
Other	6 (9%)	5 (9%)
*Treatment*		
Chemotherapy	49 (70%)	41 (71%)
Surgery	42 (60%)	39 (67%)
Radiotherapy	36 (51%)	31 (53%)
Hormone therapy	19 (27%)	18 (31%)
Immunotherapy	20 (29%)	15 (26%)
Stem cell transplantation	6 (9%)	5 (9%)
Treatment completed		
Yes	34 (49%)	28 (48%)
No	36 (51%)	30 (52%)

Results are displayed as n (%) or mean (± SD).

Abbreviations: kg·m^− 2^, kilograms per square meter; n, number of subjects

### CPET and questionnaire results

Mean CPET-VO_2peak_ at T_0_ was 18.9 ± 5.9 mL·kg^− 1^·min^− 1^, which is 62 ± 19% of the reference value for healthy Dutch persons of the same age and sex [36]. Mean time between T_0_-T_1_ was 94 ± 16 days. All included participants showed maximal voluntary exhaustion during CPET. At T_0_, n = 62 participants (89%) met at least one of the objective criteria for true maximal effort during CPET and at T_1_, n = 46 (79%). For RER_peak_ and heartrate at peak exercise (HR_peak_), no significant differences were seen between T_0_ and T_1_. Participants who completed the tests and questionnaires at both T_0_ and T_1_ showed a significant mean improvement of 1.6 mL·kg^− 1^·min^− 1^ (95%-CI 1.0‒2.3) or 8% on CPET-VO_2peak_ after completion of the exercise program. Thirty-four participants (59%) showed a relative increase of ≥ 6% in CPET-VO_2peak_ which we considered as a true improvement in aerobic capacity [34]. Body weight, VO_2AT_ during CPET, FitMáx-VO_2peak_, DASI-VO_2peak_ and VSAQ-VO_2peak_ increased significantly as well (Table 2). Most missing values were observed for DASI-VO_2peak_. Because some participants did not fill out the FitMáx question about cycling, a sub analysis was performed without the maximum cycling capacity [17]. CPET results and questionnaire-VO_2peak_ are presented in Table 2 for all participants at T_0_ (N = 70) and for the participants who completed CPET and the questionnaires at both T_0_ and T_1_ (n = 58), with corresponding change scores. The participants’ ability to complete the FitMáx on a scale from 1 to 10 is reported as well.

**Table 2:** CPET and questionnaire results

Variable	Subjects who completed CPET and questionnaires at *T*_0_ (n = 70)^a^	Subjects who completed CPET and questionnaires at T_0_ and T_1_ (n = 58)^b^		
*Anthropometric data*		**T**_**0**_	**T**_**1**_	**ΔT**_**0**_ **en T**_**1**_
Body weight (kg)	77.4 (± 15.5)	76.5 (± 15.2)	77.4 (± 15.7)	0.9 (0.2‒1.7)*
*CPET data*				
CPET-VO_2peak_ (mL·kg^− 1^·min^− 1^)	18.9 (± 5.9)	18.5 (± 5.4)	20.1 (± 5.9)	1.6 (1.0‒2.3)*
% of the reference VO_2peak_^c^	62 (± 19)	62 (± 18)	67 (± 19)	6 (4‒7)
HR_peak_ (beat·min^− 1^)	147 (± 22)	147 (± 21)	148 (± 20)	1 (-3‒5)
RER_peak_ (VCO_2_/VO_2_)	1.16 (± 0.09)	1.15 (± 0.09)	1.16 (± 0.09)	0.01 (-0.01‒0.03)
VO_2AT_ (mL·kg^− 1^·min^− 1^)	11.6 (± 3.2)	11.4 (± 2.9)	12.8 (± 3.1)	1.3 (0.7‒1.9)*
Δ Time CPET T_0_-T_1_ (days)	-	-	-	94 (89‒98)*
*Questionnaire data*				
FitMáx-VO_2peak_ (mL·kg^− 1^·min^− 1^)	23.2 (± 7.7)	22.7 (± 6.1)	24.7 (± 6.6)	1.9 (0.6‒3.3)*
FitMáx-VO_2peak_ without cycling (mL·kg^− 1^·min^− 1^)	23.8 (± 7.5)	23.5 (± 6.6)	25.3 (± 6.9)	1.8 (0.5‒3.2)*
VSAQ-VO_2peak_ (mL·kg^− 1^·min^− 1^)	19.4 (± 7.4)	18.0 (± 6.1)	21.3 (± 8.4)	3.2 (1.4‒5.1)*
DASI-VO_2peak_ (mL·kg^− 1^·min^− 1^)	22.9 (± 6.0)	22.2 (± 6.1)	25.3 (± 5.4)	3.1 (1.4‒4.8)*
*Ability to estimate FitMáx scores (1–10)*				
Walking score estimate	8 (7‒9)	8 (7‒9)	8 (7‒10)	
Stairclimbing score estimate	8 (6‒9)	8 (6‒8)	8 (7‒9)	
Cycling score estimate	5 (3‒8)	5 (3‒7)	6 (4‒8)	

Means ± SDs are presented for subjects who completed the CPET and questionnaires at T_0_

The ability to estimate the maximum capacity of walking, stairclimbing and cycling (1–10) is reported as median (interquartile range)

For subjects who completed CPET and questionnaires at T_0_ and T_1_, means ± SDs are presented for both time points with the mean difference and corresponding 95%-CI. * Statistically significant

Abbreviations: CPET, cardiopulmonary exercise test; DASI, duke activity status index; HR_peak_, heartrate at peak exercise; kg, kilograms; mL, milliliters; min, minute; n, number of subjects; RER_peak_, peak respiratory exchange ratio; VO_2AT_, oxygen uptake at the anaerobic threshold; VO_2peak_, peak oxygen uptake; VSAQ, veterans specific activity questionnaire

^*a*^ *Missing values for subjects who performed CPET and filled in questionnaires at T*_*0*_ *(n = 70): VO*_*2AT*_ *n = 1,, FitMáx n = 5, FitMáx without cycling n = 1, DASI n = 9, walking score estimate n = 2, stairclimbing score estimate n = 2, cycling score estimate n = 3*

^*b*^ *Missing values for subjects who completed CPET and questionnaires at T*_*0*_ *and T*_*1*_ *(n = 58): VO*_*2AT*_ *n = 1,, FitMáx n = 7, FitMáx without cycling n = 2, DASI n = 13, walking score estimate n = 1, stairclimbing score estimate n = 1, cycling score estimate n = 1*

^*c*^*Mean VO*_*2peak*_*calculated by prediction model for VO*_*2peak*_*of the LowLands Fitness Registry for the general Dutch population was 31.0 ± 5.8 mL·kg*^*− 1*^*·min*^*− 1*^*for this population at T*_*0*_.[36]

### Criterion validity

**Fig. 2:**
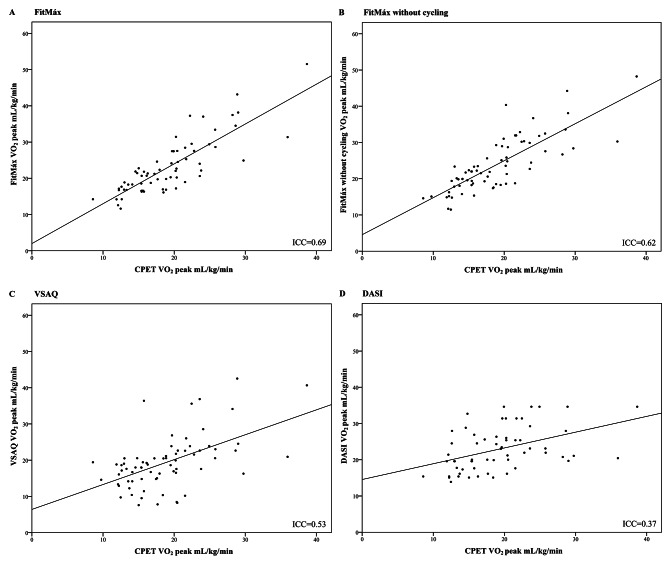
**AD**. Criterion validity with identity line for relation between questionnaire-VO_2peak_ and CPET-VO_2peak_ at T_0_. **A**) FitMáx-VO_2peak_ compared with CPET-VO_2peak_. **B**) FitMáx-VO_2peak_ without cycling compared with CPET-VO_2peak_. **C**) VSAQ-VO_2peak_ compared with CPET-VO_2peak_. **D**) DASI-VO_2peak_ compared with CPET-VO_2peak_. Abbreviations: CPET, cardiopulmonary exercise test; DASI, duke activity status index; ICC, intraclass correlation coefficient; kg, kilograms; mL, milliliters; min, minute; VO_2peak_, peak oxygen uptake; VSAQ, veterans specific activity questionnaire

**Fig. 3:**
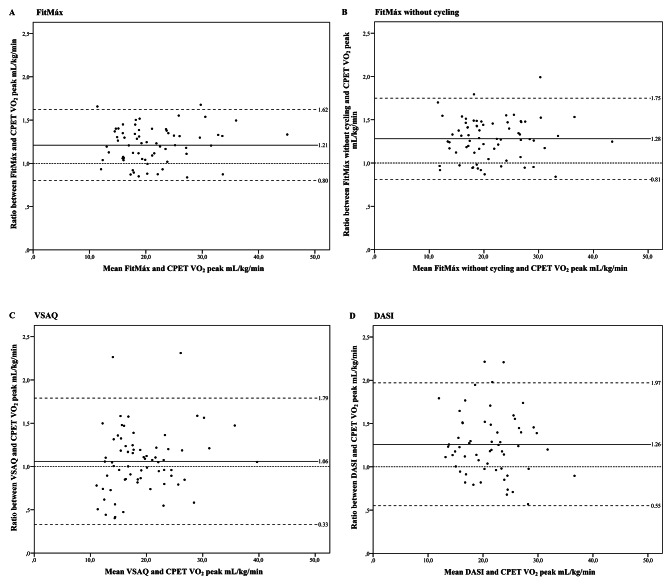
** A**-**D**. Bland-Altman plots for the agreement between questionnaire-VO_2peak_ and CPET-VO_2peak_ at T_0_. The dashed lines represent the 95%-LoA, from − 1.96 SD to + 1.96 SD. The solid line represents ratio bias and the dotted line represents the zero bias line. **A**) FitMáx-VO_2peak_ compared with CPET-VO_2peak_. **B**) FitMáx-VO_2peak_ without cycling compared with CPET-VO_2peak_. **C**) VSAQ-VO_2peak_ compared with CPET-VO_2peak_. **D**) DASI-VO_2peak_ compared with CPET-VO_2peak_. Abbreviations: CPET, cardiopulmonary exercise test; DASI, duke activity status index; kg, kilograms; mL, milliliters; min, minute; VO_2peak_, peak oxygen uptake; VSAQ, veterans specific activity questionnaire</fig>

An ICC of 0.69 (95%-CI 0.18‒0.86) was found for the agreement between CPET-VO_2peak_ and FitMáx-VO_2peak_ at T_0_. When the question about maximum cycling capacity was not included, the ICC was 0.62 (95%-CI 0.01‒0.84) for the agreement with CPET-VO_2peak_. Less agreement was found between CPET-VO_2peak_ and VSAQ-VO_2peak_ (ICC = 0.53) and CPET-VO_2peak_ and DASI-VO_2peak_ (ICC = 0.37)(Table 3). The agreement between questionnaire-VO_2peak_ and CPET-VO_2peak_ is displayed visually in Fig. 2A-D. Bland-Altman plots showed proportional bias for the agreement between CPET-VO_2peak_ and FitMáx-VO_2peak_, FitMáx-VO_2peak_ without cycling and VSAQ-VO_2peak_ (p < 0.05). For this reason, bias and 95%-LoA were reported as ratios [33]. The mean ratio of FitMáx-VO_2peak_/CPET-VO_2peak_ was 1.21 (95%-LoA 0.80–1.62), which means the FitMáx overestimated CPET-VO_2peak_ with 21% on average. The mean ratio bias was 1.28 (95%-LoA 0.81–1.75) for FitMáx-VO_2peak_ without cycling, 1.06 (95%-LoA 0.33–1.79) for VSAQ-VO_2peak_ and 1.26 (95%-LoA 0.55–1.97) for DASI-VO_2peak_. Bland-Altman plots show wider 95%-LoA for VSAQ and DASI, when compared to FitMáx. The plots for FitMáx-VO_2peak_ with and without maximum cycling capacity look similar, but the results are shifted more towards a ratio above 1 for the FitMáx-VO_2peak_ without maximum cycling capacity. SEE for the agreement between CPET-VO_2peak_ and FitMáx-VO_2peak_, FitMáx-VO_2peak_ without cycling, VSAQ-VO_2peak_ and DASI-VO_2peak_ was 3.28 mL·kg^− 1^·min^− 1^, 3.31 mL·kg^− 1^·min^− 1^, 4.95 mL·kg^− 1^·min^− 1^ and 5.46 mL·kg^− 1^·min^− 1^, respectively (Fig. 3A-D; Table 3).

**Table 3:** Agreement between CPET-VO_2peak_ and questionnaire-VO_2peak_ at T_0_

Variable	n	ICC	95%-CI	SEE	Mean ratio bias	Ratio lower 95%-LoA	Ratio upper 95%-LoA
CPET-VO_2peak_	70	n/a	n/a	n/a	n/a	n/a	n/a
FitMáx-VO_2peak_	65	0.69	0.18‒0.86*	3.28	1.21	0.80	1.62
FitMáx-VO_2peak_ without cycling	69	0.62	0.01‒0.84*	3.31	1.28	0.81	1.75
VSAQ- VO_2peak_	70	0.53	0.34‒0.68*	4.95	1.06	0.33	1.79
DASI-VO_2peak_	61	0.37	0.10‒0.59*	5.46	1.26	0.55	1.97

Number of subjects per questionnaire (n), ICC with corresponding 95%-CI, SEE and mean ratio bias with 95%-LoA are reported for the relation between CPET-VO_2peak_ and questionnaire-VO_2peak_ at T_0_. * Statistically significant

Abbreviations: CPET, cardiopulmonary exercise test; DASI, duke activity status index; ICC, intraclass correlation; n, number of subjects; n/a, not applicable; SEE, standard error of the estimate; VO_2peak_, peak oxygen uptake; VSAQ, veterans specific activity questionnaire; 95%-CI, 95% confidence interval; 95%-LoA, 95% limits of agreement

### Responsiveness

**Fig. 4:**
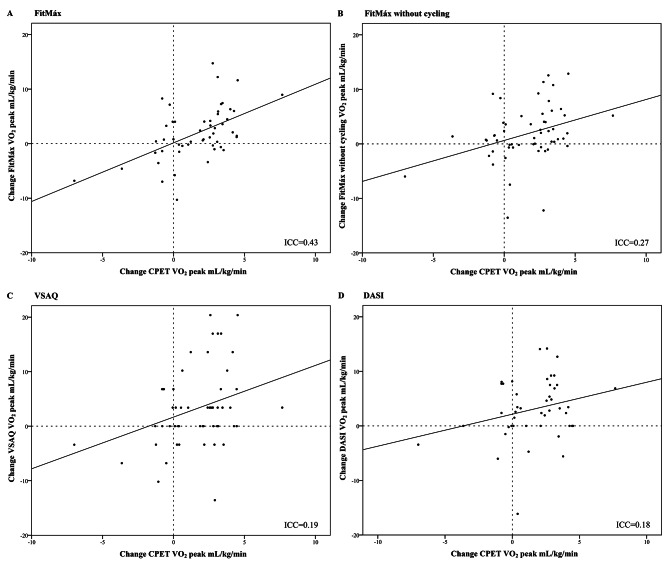
**AD**. Scatterplots for the relation between changes (Δ) in questionnaire-VO_2peak_ and CPET-VO_2peak_ from T_0_-T_1_. **A**) ΔFitMáx-VO_2peak_ compared with ΔCPET-VO_2peak_. **B**) ΔFitMáx-VO_2peak_ without cycling compared with ΔCPET-VO_2peak_. **C**) ΔVSAQ-VO_2peak_ compared with ΔCPET-VO_2peak_. **D**) ΔDASI-VO_2peak_ compared with ΔCPET-VO_2peak_ Abbreviations: CPET, cardiopulmonary exercise test; DASI, duke activity status index; ICC, intraclass correlation coefficient; kg, kilograms; mL, milliliters; min, minute; VO_2peak_, peak oxygen uptake; VSAQ, veterans specific activity questionnaire

**Fig. 5:**
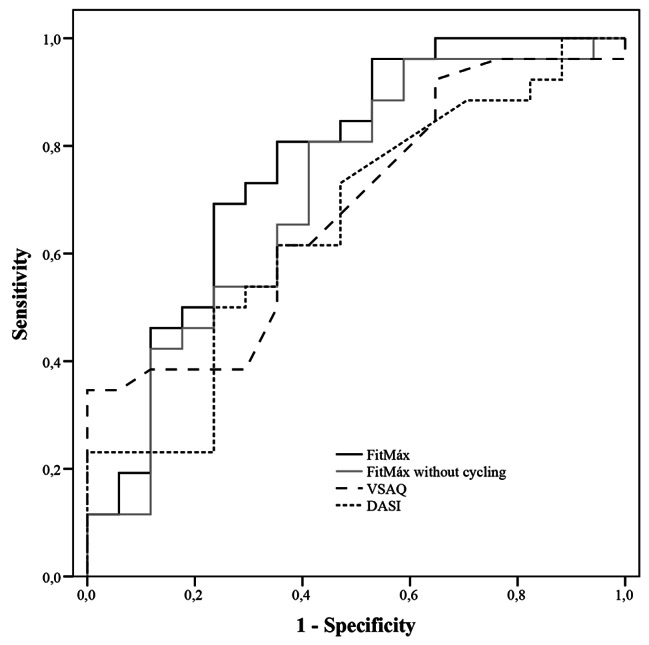
ROC-curves for the ability of questionnaires to detect a true improvement in CPET-VO_2peak_ Abbreviations: DASI, duke activity status index; ROC-curve, receiver operating characteristics curve; VSAQ, veterans specific activity questionnaire

An ICC of 0.43 (95%-CI 0.18‒0.63) was found for the agreement between individual ΔFitMáx-VO_2peak_ and ΔCPET-VO_2peak_ from T_0_ to T_1_. The ICC agreement between ΔFitMáx-VO_2peak_ without the question about maximum cycling capacity and ΔCPET-VO_2peak_ was 0.27 (95%-CI 0.00‒0.49). A lower ICC was found for the agreement between ΔCPET-VO_2peak_ and ΔVSAQ-VO_2peak_ (ICC = 0.19 95%-CI -0.06‒0.42) and the agreement between ΔCPET-VO_2peak_ and ΔDASI-VO_2peak_ (ICC = 0.18 95%-CI -0.10‒0.44) (Fig. 4A-D; Table 4). Since the responsiveness to estimate ΔCPET-VO_2peak_ was insufficient for all questionnaires, ROC analyses were performed to determine whether the questionnaires are able to detect a true improvement in CPET-VO_2peak_ (≥ 6%) with a corresponding optimal cut-off value [34] An area under the curve (AUC) of 0.77 (95%-CI 0.63–0.91) was found for FitMáx-VO_2peak_, while the FitMáx without maximum cycling capacity showed an AUC of 0.72 (95%-CI 0.59–0.86). The ROC-curve for VSAQ-VO_2peak_ and DASI-VO_2peak_ showed an AUC of 0.66 (95%-CI 0.52–0.80) and 0.64 (95%-CI 0.48–0.81), respectively (Table 4; Fig. 5). The maximum product of sensitivity and specificity was found at Δ1.0 mL·kg^− 1^·min^− 1^, for FitMáx-VO_2peak_ and Δ1.8 mL·kg^− 1^·min^− 1^ for FitMáx-VO_2peak_ without maximum cycling capacity. These values were therefore chosen as the optimal cut-off values to discriminate between improvement and no improvement in CPET-VO_2peak_. The optimal cutoff value for VSAQ-VO_2peak_ was Δ3.4 mL·kg^− 1^·min^− 1^ and Δ2.7 mL·kg^− 1^·min^− 1^ for DASI-VO_2peak_. Using the cut-off value for FitMáx-VO_2peak_, resulted in a sensitivity of 71% a specificity of 75%, a positive predictive value (PPV) of 81% and a (NPV) negative predictive value of 63%. Sensitivity, specificity, PPV and NPV for the other questionnaires are presented in Table 4.

**Table 4:** Agreement between CPET-VO_2peak_ and Questionnaire-VO_2peak_ for changes (∆) from T_0_ to T_1_

Variable	n	ICC	95%-CI	SEE	AUC	95%-CI	Cut-off value	Sens (%)	Spec (%)	PPV (%)	NPV (%)
∆CPET-VO_2peak_	58	n/a	n/a	n/a	n/a	n/a	n/a	n/a	n/a	n/a	n/a
∆ FitMáx-VO_2peak_	51	0.43	0.18‒0.63*	2.07	0.77	0.63–0.91	1.0	71	75	81	63
∆FitMáx-VO_2peak_ without cycling	56	0.27	0.00‒0.49*	2.23	0.72	0.59–0.86	1.8	61	78	80	58
∆VSAQ-VO_2peak_	58	0.19	-0.06‒0.42	2.25	0.66	0.52–0.80	3.4^◊^	62	58	68	52
∆DASI-VO_2peak_	45	0.18	-0.10‒0.44	2.40	0.64	0.48–0.81	2.7	62	63	70	55

Number of subjects per variable (n) and ICC with corresponding 95%-CI are reported for the relation between ΔCPET-VO_2peak_ and Δquestionnaire-VO_2peak_ from T_0_ to T_1_. * Statistically significant ^◊^The cut-off value for VSAQ-VO_2peak_ is also the smallest improvement (which is ~ equal to 1.0 MET) that could be measured with VSAQ [12].

Abbreviations: AUC, area under the curve; CPET, cardiopulmonary exercise test; DASI, duke activity status index; ICC, intraclass correlation; n, number of subjects; n/a, not applicable; NPV, negative predictive value; PPV, positive predictive value; SEE, standard error of the estimate; Spec, specificity; Sens, sensitivity; VO_2peak_, peak oxygen uptake; VSAQ, veterans specific activity questionnaire; 95%-CI, 95% confidence interval

## Discussion

In this study among cancer survivors who participated in a 10-week exercise program, we evaluated the criterion validity of three questionnaire and found a moderate agreement between FitMáx-VO_2peak_ and CPET- VO_2peak_. Agreement between CPET-VO_2peak_ and VSAQ-VO_2peak_ was moderate as well, but lower compared to FitMáx-VO_2peak_, while the DASI-VO_2peak_ showed poor agreement. This implies that the criterion validity of the DASI to evaluate aerobic capacity was insufficient. The criterion validity of the FitMáx and the VSAQ to estimate aerobic capacity is acceptable on group level, but limited to estimate CPET-VO_2peak_ in individuals [19].

Initial Bland-Altman analysis showed proportional bias, indicating that mean differences between questionnaire-VO_2peak_ and CPET-VO_2peak_ with corresponding 95%-LoA, are dependent on the size of the CPET-VO_2peak_ values. This is not surprising, since higher measurement errors are expected for higher values of CPET-VO_2peak_ [34]. For the latter reason, Bland-Altman analyses were performed using ratios instead of differences between questionnaire-VO_2peak_ and CPET-VO_2peak_, which showed an overestimation of CPET-VO_2peak_ for all questionnaires [33]. Mean ratio bias for FitMáx-VO_2peak_ (+ 21%) was smaller compared to DASI-VO_2peak_ (+ 26%), but larger compared to VSAQ-VO_2peak_ (+ 6%). However, 95%-LoA for VSAQ-VO_2peak_ were wider compared to those for FitMáx-VO_2peak_. This could be explained by larger measurement errors for VSAQ-VO_2peak_ in both directions, while FitMáx and DASI overestimated CPET-VO_2peak_ in most cases.

The moderate agreement found between questionnaire-VO_2peak_ and CPET-VO_2peak_ is in line with previous research, which showed discrepancies between patient-reported functional capacity and measured VO_2peak_ [13, 37]. A recent study of Meijer et al., reported higher values for the agreement between CPET-VO_2peak_ and FitMáx-VO_2peak_, DASI-VO_2peak_ and VSAQ-VO_2peak_. On the other hand, SEE for FitMáx-VO_2peak_ and VSAQ-VO_2peak_ were smaller in the current study, compared to the previous study, indicating more accurate predictions of CPET-VO_2peak_ [17]. It was not possible to compare Bland-Altman results with previous studies, because ratios were used instead of absolute values in the current study. In the original studies about the development of DASI and VSAQ, higher correlation coefficients between estimated and measured aerobic capacity were found, but the populations and research methods differed substantially from our study and both studies were performed more than 25 years ago [11, 12]. Low ICC values for the agreement between questionnaire-VO_2peak_ and CPET-VO_2peak_ at T_0_ in the current study, could be explained by the small range in VO_2peak_ values [38]. The current study population had a relatively low aerobic capacity (62% of predicted) and the population was more homogeneous compared to the original FitMáx study [17]. The fact that participants in the current study reached lower fitness levels compared to participants in the original FitMáx study (in which the questionnaire and its prediction model were developed), may have influenced the performance of the questionnaire as well. It can be expected that estimating physical abilities is easier when someone is fitter and reaches higher physical activity levels in daily life or even in sports. For patients who are mainly sedentary, it might be more difficult to estimate their physical abilities. Moreover, it could be questioned whether the question about cycling of the FitMáx is appropriate for the current study population. The area of the MUMC + is hilly, making it difficult for elderly to cycle on a regular bike, especially after receiving cancer treatment. When patients did not cycle regularly, or cycled on an electronic bike, it may have been hard for them to answer the FitMáx question about maximum cycling capacity. This is in line with the fact that participants rated their ability to complete the FitMáx question about cycling with a median of 5 at T_0_ and 6 at T_1_, which is lower compared to the other two questions about walking and stair climbing.

All three questionnaires showed poor responsiveness to measure ΔCPET-VO_2peak_ in the current study population. This could be explained by the increased measurement error that comes along with repeated testing and by the little variability in data as well [20, 21, 38]. However, ROC analysis showed that FitMáx-VO_2peak_ was sufficiently responsive to detect a true improvement in CPET-VO_2peak_ (AUC 0.77), when using the optimal cut-off value of 1.0 mL·kg^− 1^·min^− 1^ [34]. This was also the case for the FitMáx-VO_2peak_ without the question about maximum cycling capacity (AUC 0.72 with a cut-off value of 1.8 mL·kg^− 1^·min^− 1^). The AUC for DASI-VO_2peak_ (0.64) and VSAQ-VO_2peak_ (0.66) were insufficient to detect improvement, and therefore it is not recommended to use these questionnaires to monitor changes in aerobic capacity.

Comparing the current study results to a previous study in which a mean change of 2.0 ± 2.3 mL·kg^− 1^·min^− 1^ was found after a 10-week exercise program as part of multidisciplinary oncology rehabilitation in MUMC+, larger improvements in VO_2peak_ were expected [23]. This could be explained by the fact that the training stimulus in the current study was not given as intended, due to COVID-19. Because of this pandemic, patients were allowed to train only once a week instead of twice and exercise training took place in smaller groups of four instead of eight patients. In order to avoid a long waiting list, the training frequency was reduced. The smaller improvement may have led to less variability in ΔVO_2peak_ from T_0_ to T_1_, which could explain low ICC values for responsiveness [38]. Results for responsiveness could not be compared with literature, because no previous studies were conducted on this matter.

Comparing the results for the different questionnaires, we can conclude that values for criterion validity and responsiveness of the FitMáx-VO_2peak_ are better compared to VSAQ-VO_2peak_ and DASI-VO_2peak_, in cancer survivors participating in an exercise program. FitMáx-VO_2peak_ was less accurate without the question for maximum cycling capacity, yet superior to the DASI and VSAQ.

### Strengths of the current study

This is the first study to investigate the responsiveness of self-reported questionnaires to estimate ΔVO_2peak_. The direct comparison of the criterion validity and responsiveness of three different self-reported questionnaires, with CPET-VO_2peak_ as criterion standard measure, was a strength of this study. Since both measurements and the exercise training were part of usual care, the current study results can easily be translated into daily care in oncology rehabilitation in the Netherlands. Besides, we included patients who did and did not complete medical treatment yet, resulting in a variation of ΔCPET-VO_2peak_ in both directions, which is ideal to study the responsiveness of a measurement [5, 21]. Another strength of the study was blinding of participants and researchers for test outcomes to avoid bias.

### Limitations of the current study

A limitation was the fact that the DASI was often not completed. A possible explanation is the use of twelve dichotomous questions also including some activities which are difficult to recognize for the general Dutch population, such as playing basketball. In the absence of only one answer the DASI-VO_2peak_ could not be calculated. This suggests that the usability of the DASI is limited in this population. The fact that true maximum effort (according to objective criteria) was not reached during all CPETs, could be seen as a limitation as well. However, these findings are in agreement with previous studies, which reported that maximal effort criteria are often not reached in cancer survivors [23, 39]. Besides, it can be expected that these participants are also unable to reach and estimate their maximum capacity of walking, stairclimbing, cycling and other daily tasks, as described in the self-reported questionnaires. Since mean RER_peak_ and HR_peak_ were similar at T_0_ and T_1_, it is not expected that the delivered effort affected the study results. Another limitation was the fact that the study population is quite specific (79% women and in general low fitness) so results may not be generalizable to other patients with cancer. Validity and responsiveness for male cancer survivors could differ from the current study results, especially because VO_2peak_ is sex-dependent. Also the cancer type and treatment may influence the relationship between questionnaire-VO _2peak_ and CPET-VO_2peak_. For instance, breast surgery and breast radiation may cause limitations in certain activities mentioned in the DASI and VSAQ that include the upper body (i.e. lifting weights). More research is needed in a population with a better distribution of sex, cancer type, treatment and more variation in level of aerobic capacity. Also, research on the responsiveness of PROMs to measure deterioration in VO_2peak_ would be of additional value, since the current study focused on improvement. Monitoring deterioration in VO_2peak_ would be useful during intensive cancer treatment, like chemotherapy. In this case, rehabilitation can be started as soon as deterioration in VO_2peak_ is noted. Besides, PROMs for estimating aerobic capacity could potentially be improved in the future, by using computerized adaptive test (CAT) methods. CAT methods enable PROMs to be adapted to individual patients while maintaining direct comparability of the scores [40, 41]. Based on the patient’s previous answers, a computer program personalizes the next questions, in order to obtain precise information in an efficient manner. A CAT version of the FitMáx, could personalize questions on physical fitness for patients with different diagnoses of cancer, different treatment modalities and different fitness levels, which could potentially lead to more precise estimations of VO _2peak_and better values of validity and responsiveness.

### Clinical relevance

Results of the current study show that the FitMáx is sufficiently valid to estimate aerobic capacity on group level and could be used to detect improvement using a cutoff value of 1.0 mL.kg^− 1^.min^− 1^. The advantage of such a questionnaire is the possibility to monitor aerobic capacity over time with repeated assessments at low cost. When choosing self-reported questionnaires to evaluate aerobic capacity in cancer survivors, it can be recommended to use FitMáx above the DASI and VSAQ, since this recently developed questionnaire showed better criterion validity, and a responsiveness above the 0.70 AUC threshold. However, some results should be interpreted with caution, since values for criterion validity and responsiveness were still suboptimal, and it should be kept in mind that the FitMáx overestimates with on average 21% in this population [25]. Moreover, CPET is also used to determine the underlying cause of exercise limitations and contra-indications for physical exercise [9]. Therefore, FitMáx should not be considered as a full replacement for CPET, but rather an alternative tool to be used in clinical or research settings where exercise testing is not feasible or necessary. In cancer survivors with increased cardiovascular risks, such as pre-existing cardiovascular disease, treatment with cardiotoxic chemotherapy and left-sided chest radiation, performing CPET should still be recommended [42]. An online platform (www.fitmaxquestionnaire.com) was developed, to enable healthcare professionals and researchers in using the FitMáx. The online platform provides up-to-date information about the questionnaire and research projects. More information about the research group, hospital and FitMáx can be found on https://www.mmc.nl/english/fitmax/.

## Conclusion

The population specific criterion validity and responsiveness of the self-reported FitMáx-VO_2peak_ are better compared to VSAQ-VO_2peak_ and DASI-VO_2peak_, in cancer survivors who participated in an exercise program as part of multidisciplinary rehabilitation. The FitMáx is sufficiently valid to estimate CPET-VO_2peak_ in cancer survivors on group level, but overestimates with on average 21%. The responsiveness of the FitMáx to measure absolute changes in CPET-VO_2peak_ was poor, but the questionnaire is able to detect whether aerobic capacity improved when using a cutoff value of only 1.0 mL.kg^− 1^.min^− 1^. Therefore, the self-reported FitMáx can be used to estimate and monitor aerobic capacity in cancer survivors, but results should be interpreted with caution on absolute values, since the agreement with the criterion standard is limited. Refinements of the questionnaire and the prediction model will be made in the future potentially leading to a further optimization of the validity and responsiveness.

## Data Availability

The datasets used and/or analysed during the current study are available from the corresponding author on reasonable request.

## Abbreviations

AUC: Area Under the Curve
BMI: Body Mass Index
COSMIN: Consensus-Based Standards for the Selection of Health Measurement Instruments
CPET: Cardiopulmonary Exercise Testing
CPET-VO_2peak_: VO_2peak_ measured during CPET
DASI: Duke Activity Status Index
DASI-VO_2peak_: VO_2peak_ estimated by the Duke Activity Status Index
FitMáx: FitMáx©-questionnaire
FitMáx-VO_2peak_: VO_2peak_ estimated by the FitMáx©-questionnaire
HR_peak_: Heartrate at peak exercise
ICC: Intraclass Correlation Coefficient
kg: Kilograms
kg·m^− 2^: kg per square meter
MET: Metabolic Equivalent of Task
min: Minute
mL: Millilitres
MUMC +: Maastricht University Medical Center
n: Number of subjects
NPV: Negative Predictive Value
PPV: Positive Predictive Value
PROM: Patient-reported Outcome Measure
Questionnaire-VO_2peak_: VO_2peak_ estimated by the questionnaires
r: Pearson correlation coefficient
RER: Respiratory Exchange Ratio
RER_peak_: Respiratory Exchange Ratio at peak exercise
ROC-curve: Receiver Operating Characteristics Curve
SD: Standard Deviation
SEE: Standard Error of the Estimate
SPSS: Statistical Package for the Social Sciences
T_0_: Measurement moment at baseline
T_1_: Measurement moment after ~ 10 weeks
VO_2AT_: Oxygen uptake at the anaerobic threshold
VO_2peak_: Peak oxygen uptake
VSAQ: Veterans Specific Activity Questionnaire
VSAQ-VO_2peak_: VO_2peak_ estimated by the Veterans Specific Activity Questionnaire
95%-CI: 95% Confidence Interval
95%-LoA: 95% Limits of Agreement
Δ: Change
